# Longitudinal change of six common inflammatory cytokines and their relationship to anxiety, depression, and cognitive impairment in acute ischemic stroke patients

**DOI:** 10.1590/1414-431X2023e13025

**Published:** 2023-10-20

**Authors:** Qun Shi, Ruorui Li, Zhiling Qu, Yonghua Lang, Guiling Sheng, Jiajia Ning, Wanli Zhang

**Affiliations:** 1 Department of Endocrinology, Laizhou People's Hospital, Laizhou, China; 2 Department of Respiratory Medicine, Laizhou People's Hospital, Laizhou, China; 3 Department of Obstetrics, Laizhou People's Hospital, Laizhou, China; 4 Department of Neurology, Laizhou People's Hospital, Laizhou, China; 5 Department of Cardiology, Laizhou People's Hospital, Laizhou, China; 6 Department of Neurology, Laizhou People's Hospital, Laizhou, China

**Keywords:** Acute ischemic stroke, Inflammatory cytokines, Anxiety, Depression, Cognitive impairment

## Abstract

Inflammatory cytokines are known to be involved in acute ischemic stroke (AIS), while the relationship of multiple inflammatory cytokines with mental disorders in AIS is less reported. This research intended to explore the longitudinal variation of common inflammatory cytokines and their correlation with anxiety, depression, and cognitive impairment in AIS patients. Six inflammatory cytokines were detected by enzyme-linked immunosorbent assay among 175 AIS patients at admission (baseline) and on the day (D)1, D3, and D7 after admission. Anxiety, depression, and cognition were evaluated using the Hospital Anxiety and Depression Scale and Mini-Mental State Examination at discharge, respectively. Anxiety, depression, and cognitive impairment rates were 32.6, 39.4, and 19.4%, respectively. Tumor necrosis factor (TNF)-α, interleukin (IL)-1β, IL-6, IL-8, and IL-17A increased from baseline to D1, then decreased from D1 to D7 (all P<0.001), while IL-10 presented an opposite trend (P<0.001). Interestingly, TNF-α on D1 and D3, IL-6 on D3, IL-8 on D3 and D7, and IL-17A on D1, D3, and D7 correlated with higher anxiety rate (all P<0.05). TNF-α on D1, D3, and D7, IL-8 at baseline, D1, D3, and D7, IL-17A on D1 and D7 correlated with increased depression rate (all P<0.05). In addition, IL-1β on D1 and IL-17 at baseline, D1, D3, and D7 correlated with elevated cognitive-impairment rate (all P<0.05). Inflammatory cytokines were dysregulated after disease onset, and their longitudinal change correlated with psychological issues in AIS patients.

## Introduction

Acute ischemic stroke (AIS), a cerebral vascular disease, is one of the main causes of neurological death worldwide (1,2). AIS also results in permanent cognitive and functional impairment, which induces a huge social burden in the world (3-5). To date, the strategy of AIS therapy (such as revascularization therapy, neuroprotective treatment, intravenous thrombolysis, anticoagulation treatment, etc.) has achieved great progress, which increases the survival of AIS patients to some extent (6-8). Nevertheless, approximately 33% of AIS patients face psychological issues such as anxiety, depression, and cognitive impairment, which greatly worsen their quality of life and are related to unsatisfactory prognoses (9,10). Therefore, the exploration of factors linked to mental disorders is needed for the benefit of these patients.

It is well-known that inflammatory cytokines take part in the development of AIS, which damages neurons and accelerates atherosclerosis (11,12). Interestingly, inflammatory cytokines are also related to psychological complications in patients with cardiac-cerebral vascular diseases (13,14). For example, increased tumor necrosis factor-α (TNF‐α) and interleukin (IL)‐1β, IL-6, and IL-17A are linked to the occurrence of anxiety and depression in coronary heart disease patients (14). Moreover, TNF‐α is positively correlated with depression in myocardial infarction patients (13). Importantly, it also has been reported that TNF-α, IL-1β, and IL-17A are correlated with increased severity of mental disorders in AIS patients (15). Nevertheless, previous studies only explore limited inflammatory cytokines in AIS, and the correlation between longitudinal change of multiple inflammatory cytokines with psychological complications in AIS is unclear.

Hence, the objective of this research was to discover the vertical variation of inflammatory cytokines after disease onset and its correlation with anxiety, depression, and cognitive impairment among AIS patients.

## Material and Methods

### Patients

Between February 2021 and September 2022, a total of 175 AIS patients admitted to Laizhou People's Hospital were enrolled. The inclusion criteria were: 1) aged ≥18 years; 2) diagnosed as first-episode AIS; and 3) willing to provide peripheral blood samples for analysis and willing to assess anxiety, depression, and cognitive impairment when discharged. The exclusion criteria were: 1) diagnosed as hemorrhage stroke; 2) complicated with severe infection; 3) had autoimmune diseases; 4) clinically diagnosed with anxiety, depression, or cognitive impairment prior to admission; 5) pregnancy or lactation. The current study was approved by the Hospital Ethics Committee. All patients or their legal guardians provided informed consent.

### Sample collection

On the day of admission (baseline) and day 1 (D1), day 3 (D3), and day 7 (D7) after admission, peripheral blood samples of AIS patients were collected. Then, serum samples were isolated and stored for detection of inflammatory cytokines by enzyme-linked immunosorbent assay (ELISA).

### Detection of inflammatory cytokines

The corresponding ELISA kits were used to detect serum inflammatory cytokines including TNF-α (Invitrogen, KAC1751, USA, sensitivity of 3 pg/mL, range of 15-1500 pg/mL), IL-1β (Invitrogen, BMS224HS, sensitivity of 0.05 pg/mL, range of 0.16-10.0 pg/mL), IL-6 (Invitrogen, EH2IL6, sensitivity <1 pg/mL, range of 10.24-400 pg/mL), IL-8 (Invitrogen, KAC1301, sensitivity of 0.7 pg/mL, range of 7-750 pg/mL), IL-10 (Invitrogen, KHC0101, sensitivity <1 pg/mL, range of 7.8-500 pg/mL), and IL-17A (Invitrogen, KAC1591, sensitivity of 2 pg/mL, range of 15-1000 pg/mL). The procedures of ELISA assay were in accordance with the guideline from the manufacturer.

### Assessment of psychological issues

On the day of discharge, all patients were required to assess their anxiety, depression, and cognitive impairment using the Hospital Anxiety and Depression Scale for anxiety (HADS-A) and depression (HADS-D), and Mini-Mental State Examination (MMSE) score, respectively. Patients were considered to have anxiety or depression if their HADS-A or HADS-D score was >7; and cognitive impairment was considered if their MMSE score was <27 (16). In addition, patients with MMSE score <20 (which might affect the assessment of HADS scores) when discharged or those that died after admission were not included in the analysis.

### Statistics

SPSS (ver. 24, IBM, USA) and Graphpad Prism (ver. 7, Graphpad Software, USA) were used for statistical analysis and figure plotting, respectively. Comparison of inflammatory cytokines among different time points was conducted with Friedman test. Comparison of inflammatory cytokines between groups was conducted with Mann Whitney U test. A P value <0.05 was considered as statistical significance.

## Results

### Study flow

**Figure 1:**
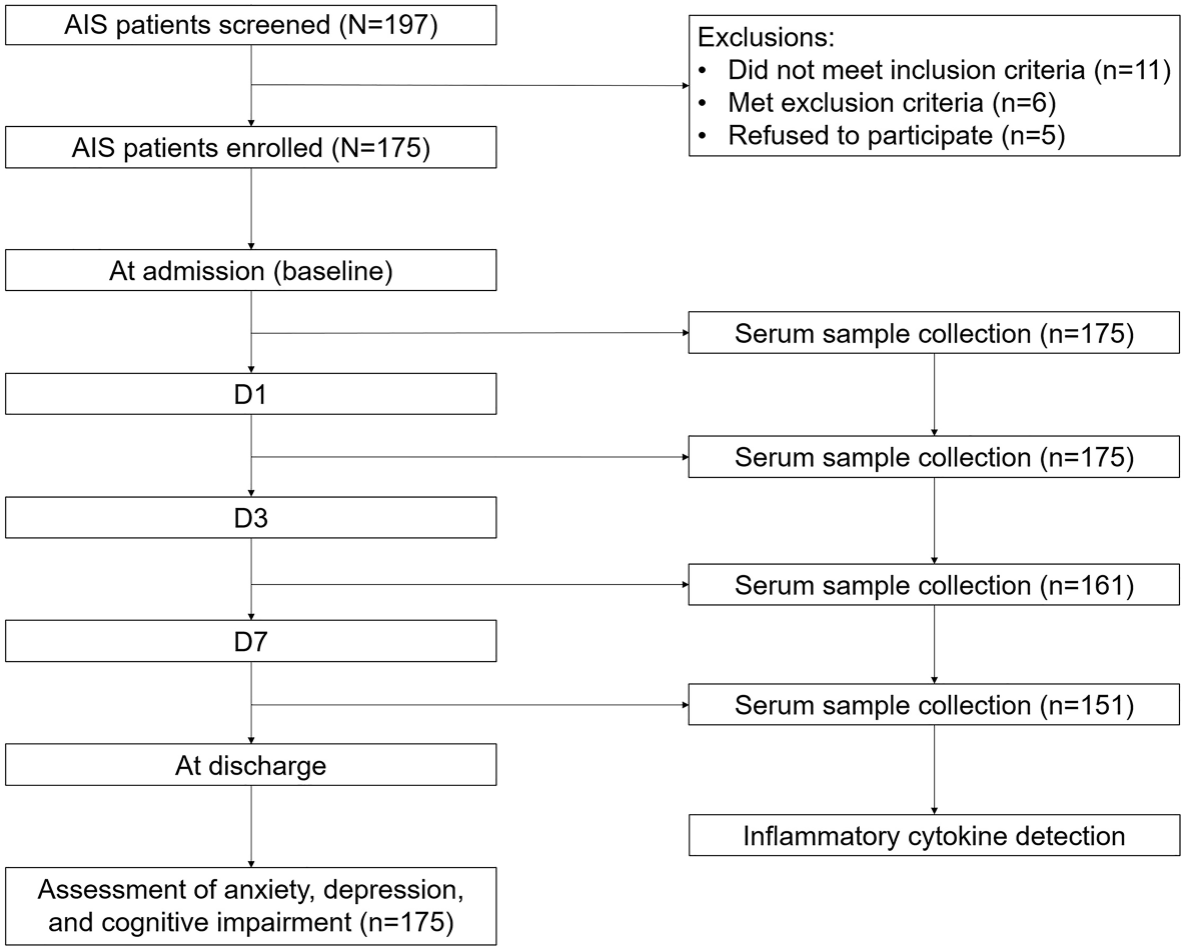
Study flow chart. AIS: acute ischemic stroke.

A total of 197 AIS patients were screened, and 22 patients were excluded. Then, 175 patients were enrolled, from whom serum sample collection and inflammatory cytokines detection were conducted at admission and on D1, D3, D7, and at discharge. Subsequently, assessments of psychological issues were conducted at discharge. More detailed information is presented in Figure 1.

### AIS patients' features

The mean age of the 175 AIS patients was 68.7±9.5 years; 57 (32.6%) were female and 118 (67.4%) were male. Additionally, 94 (53.7%) patients had a history of smoking, 150 (85.7%) patients, hypertension, 77 (44.0%), hyperlipidemia, 42 (24.0%), diabetes mellitus, and 32 (18.3%) patients had chronic kidney disease. In addition, 145 (82.9%) and 30 (17.1%) patients underwent thrombolysis and mechanical embolectomy, respectively (Table 1).

**Table 1:** Characteristics of acute ischemic stroke (AIS) patients.

Item	AIS patients (n=175)
Age (years)	68.7±9.5
Gender	
Female	57 (32.6)
Male	118 (67.4)
Body mass index (kg/m^2^)	24.65±2.60
History of smoking	
No	81 (46.3)
Yes	94 (53.7)
Hypertension	
No	25 (14.3)
Yes	150 (85.7)
Hyperlipidemia	
No	98 (56.0)
Yes	77 (44.0)
Diabetes mellitus	
No	133 (76.0)
Yes	42 (24.0)
Chronic kidney disease	
No	143 (81.7)
Yes	32 (18.3)
NIHSS score	8.6±4.8
Duration since symptoms to admission (h)	5.2±2.2
Treatment	
Thrombolysis	145 (82.9)
Mechanical embolectomy	30 (17.1)

Data are reported as means±SD or number (%). NIHSS: National Institutes of Health Stroke Scale.

### Psychological issues at discharge of AIS patients

The mean HADS-A score was 7.3±2.6 and 57 (32.6%) patients had anxiety. Furthermore, the mean HADS-D score was 7.5±2.6 and 69 (39.4%) patients had depression. Moreover, the mean MMSE score was 27.4±1.6 and there were 34 (19.4%) patients with cognitive impairment (Table 2).

**Table 2:** Anxiety, depression, and cognitive impairment at discharge in acute ischemic stroke (AIS) patients.

Item	AIS patients (n=175)
HADS-A score	7.3±2.6
Anxiety	
No	118 (67.4)
Yes	57 (32.6)
HADS-D score	7.5±2.6
Depression	
No	106 (60.6)
Yes	69 (39.4)
MMSE score	27.4±1.6
Cognitive impairment	
No	141 (80.6)
Yes	34 (19.4)

Data are reported as means±SD or number (%). HADS: Hospital Anxiety and Depression Scale; MMSE: Mini-Mental State Examination.

### Change of inflammatory cytokines in AIS patients

**Figure 2:**
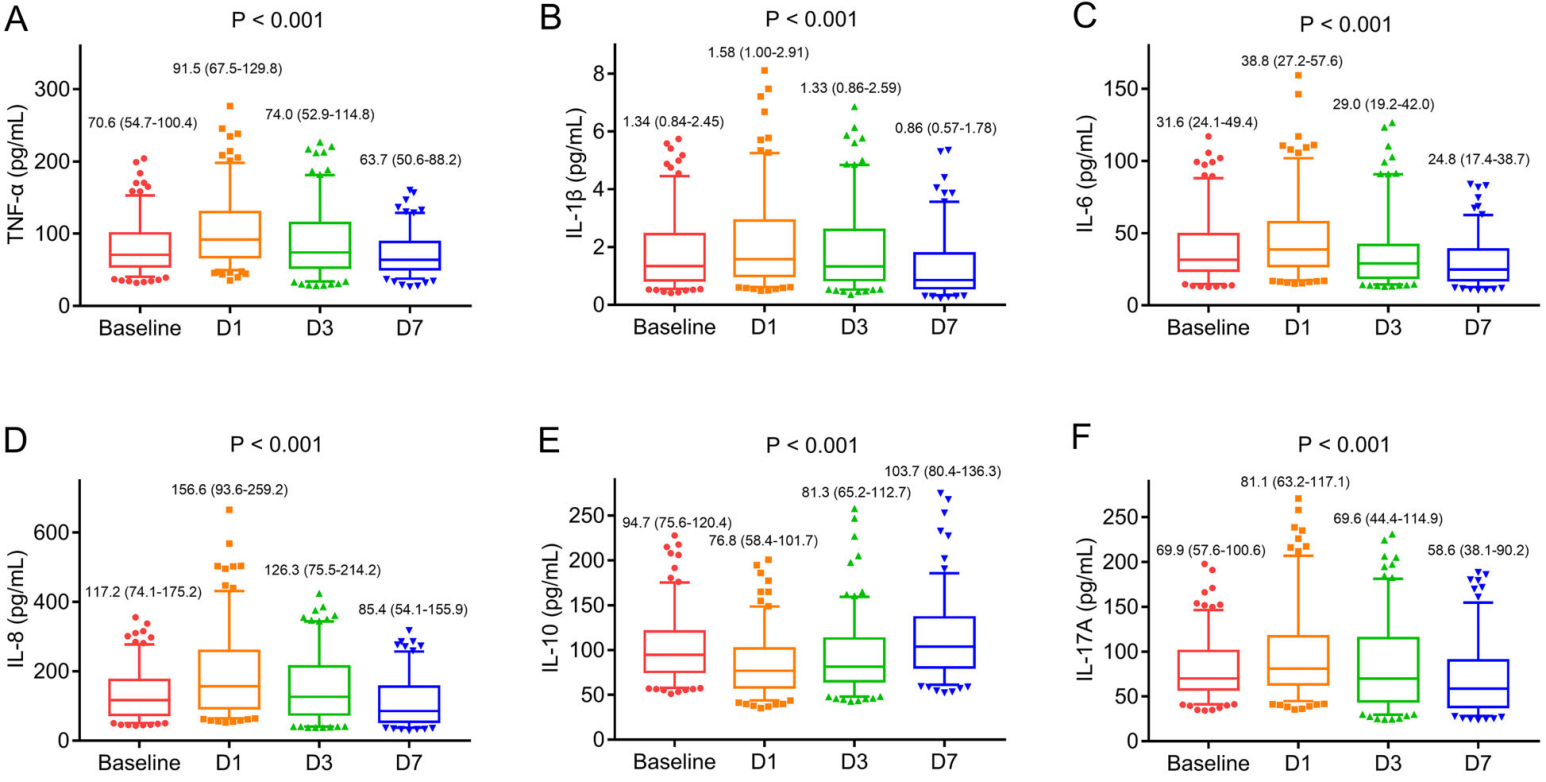
Inflammatory cytokines in acute ischemic stroke patients at different time points. Change of tumor necrosis factor (TNF)-α (**A**), interleukin (IL)-1β (**B**), IL-6 (**C**), IL-8 (**D**), IL-10 (**E**), and IL-17A (**F**) at baseline, D1, D3, and D7. Data are reported as median and interquartile range (Friedman test).

An increase from baseline to D1 was found in TNF-α (Figure 2A), IL-1β (Figure 2B), IL-6 (Figure 2C), and IL-8 (Figure 2D), and a decrease was found from D1 to D7 (all P<0.001). However, a decrease of IL-10 was seen from baseline to D1 and afterward IL-10 increased from D1 and D7 (P<0.001) (Figure 2E). Additionally, IL-17A was upregulated from baseline to D1 and declined from D1 to D7 (P<0.001) (Figure 2F).

### Correlation between inflammatory cytokines and anxiety in AIS patients at discharge

**Figure 3:**
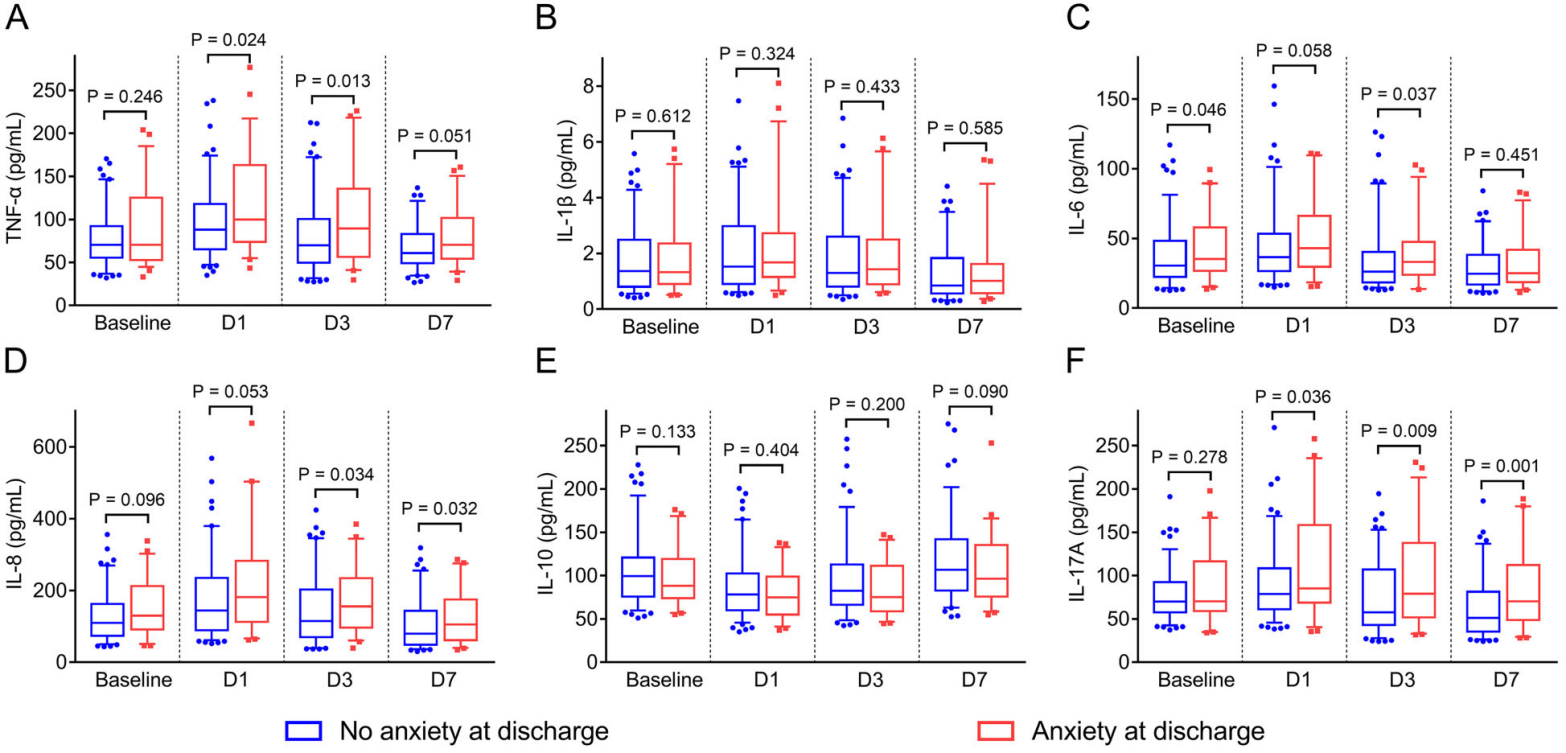
Comparison of tumor necrosis factor (TNF)-α (**A**), interleukin (IL)-1β (**B**), IL-6 (**C**), IL-8 (**D**), IL-10 (**E**), and IL-17A (**F**) at baseline, D1, D3, and D7 in acute ischemic stroke patients with anxiety compared to those without anxiety. Data are reported as median and interquartile range (Mann Whitney U-test).

Anxiety was positively associated with TNF-α on D1 (P=0.024) and D3 (P=0.013) (Figure 3A). Nevertheless, anxiety was not correlated to IL-1β at any time point (all P>0.05) (Figure 3B). A positive correlation was found between anxiety and IL-6 at baseline (P=0.046) and on D3 (P=0.037) (Figure 3C). Anxiety was also correlated to IL-8 on D3 (P=0.034) and D7 (P=0.032) (Figure 3D). Nevertheless, anxiety was not correlated to IL-10 at any time point (all P>0.05) (Figure 3E). Furthermore, anxiety was positively correlated to IL-17A on D1 (P=0.036), D3 (P=0.009), and D7 (P=0.001) (Figure 3F).

### Correlation between inflammatory cytokines and depression in AIS patients at discharge

**Figure 4:**
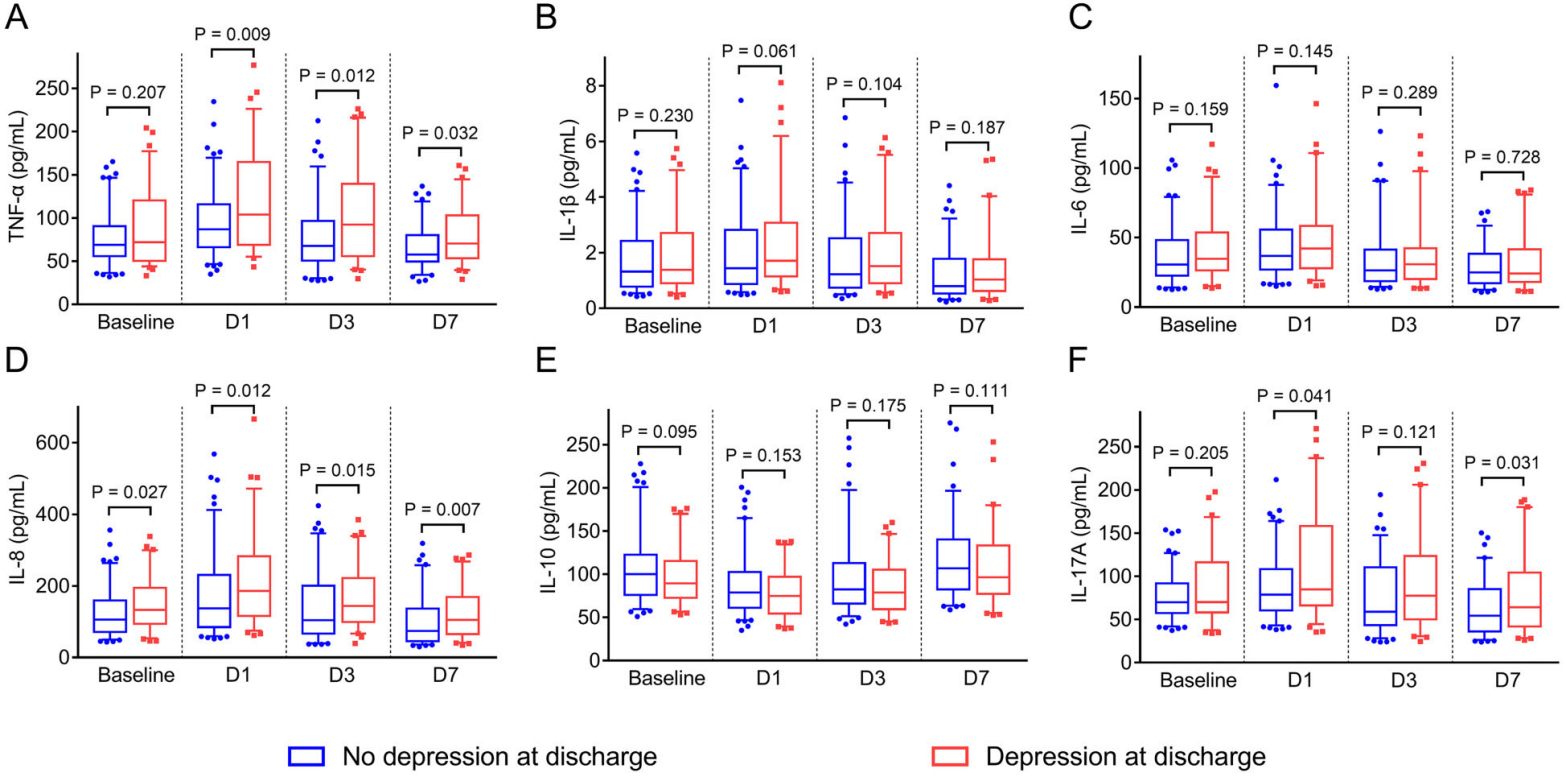
Comparison of tumor necrosis factor (TNF)-α (**A**), interleukin (IL)-1β (**B**), IL-6 (**C**), IL-8 (**D**), IL-10 (**E**), and IL-17A (**F**) at baseline, D1, D3, and D7 in acute ischemic stroke patients with depression compared to those without depression. Data are reported as median and interquartile range (Mann Whitney U-test).

Depression was positively correlated to TNF-α on D1 (P=0.009), D3 (P=0.012), and D7 (P=0.032) (Figure 4A). However, no correlation was found between depression and IL-1β (Figure 4B) or IL-6 (Figure 4C) at any time point (all P>0.05). Furthermore, depression was positively correlated to IL-8 at baseline (P=0.027) and on D1 (P=0.012), D3 (P=0.015), and D7 (P=0.007) (Figure 4D). Nevertheless, depression was not associated with IL-10 at any time point (all P>0.05) (Figure 4E). A positive correlation was found for depression with IL-17A on D1 (P=0.041) and D7 (P=0.031) (Figure 4F).

### Correlation between inflammatory cytokines and cognitive impairment in AIS patients at discharge

**Figure 5:**
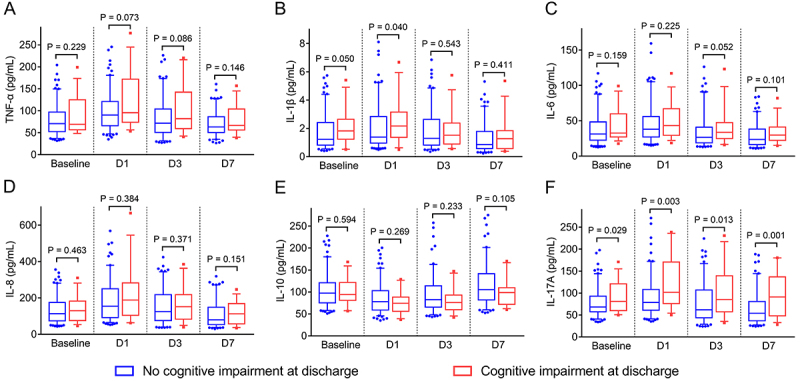
Comparison of tumor necrosis factor (TNF)-α (**A**), interleukin (IL)-1β (**B**), IL-6 (**C**), IL-8 (**D**), IL-10 (**E**), and IL-17A (**F**) at baseline, D1, D3, and D7 in acute ischemic stroke patients with cognitive impairment compared to those without cognitive impairment. Data are reported as median and interquartile range (Mann Whitney U test).

Cognitive impairment was not correlated with TNF-α at any time point (all P>0.05) (Figure 5A). However, cognitive impairment was positively correlated to IL-1β at D1 (P=0.040) (Figure 5B). Nevertheless, cognitive impairment was not correlated to IL-6 (Figure 5C), IL-8 (Figure 5D), or IL-10 (Figure 5E) at any time point (all P>0.05). A positive correlation was found between cognitive impairment and IL-17A at baseline (P=0.029) and D1 (P=0.003), D3 (P=0.013), and D7 (P=0.001) (Figure 5F).

### Correlation of inflammatory cytokine changes with psychological issues in AIS patients at discharge

IL-6 change from baseline to D7 was positively correlated to anxiety (P=0.004) and depression (P=0.027) at discharge. Meanwhile, IL-17A change from baseline to D7 was negatively associated to anxiety (P=0.001) and cognitive impairment (P=0.017) at discharge. Other inflammatory cytokine changes were not correlated to psychological issues (all P>0.05) (Table 3).

**Table 3:** Correlation of inflammatory cytokine changes with psychological issues in acute ischemic stroke (AIS) patients.

Items	Inflammatory cytokine changes (baseline-day 7)					
	TNF-α level (pg/mL)	IL-1β level (pg/mL)	IL-6 level (pg/mL)	IL-8 level (pg/mL)	IL-10 level(pg/mL)	IL-17A level (pg/mL)
AIS patients	7.0 (-1.5, 19.3)	0.4 (0.1, 0.8)	5.9 (2.1, 12.5)	22.2 (7.1, 49.9)	-4.2 (-22.8, 9.3)	12.4 (2.0, 27.0)
Anxiety at discharge						
No	7.9 (-0.6, 19.2)	0.3 (0.1, 0.8)	4.4 (1.2, 10.7)	23.7 (7.9, 48.9)	-4.9 (-22.8, 9.0)	18.5 (6.0, 30.5)
Yes	3.2 (-4.8, 22.2)	0.4 (0.1, 0.9)	9.6 (3.6, 14.6)	20.3 (-0.2, 61.2)	-0.6 (-22.5, 13.1)	8.4 (-6.5, 18.5)
P value	0.187	0.384	0.004	0.674	0.902	0.001
Depression at discharge						
No	9.6 (-0.6, 20.1)	0.3 (0.1, 0.8)	4.5 (1.2, 11.1)	25.7 (11.3, 48.1)	-3.4 (-24.8, 10.0)	15.4 (4.0, 28.8)
Yes	3.9 (-4.4, 14.9)	0.4 (0.1, 0.9)	7.6 (3.2, 13.8)	19.5 (-1.1, 60.4)	-5.8 (-20.3, 9.4)	9.1 (-3.8, 25.9)
P value	0.068	0.555	0.027	0.525	0.810	0.083
Cognitive impairment at discharge						
No	6.9 (-1.7, 19.0)	0.3 (0.1, 0.7)	5.1 (1.9, 12.0)	24.9 (6.7, 50.5)	-5.4 (-25.9, 9.2)	15.0 (4.5, 29.0)
Yes	8.3 (-0.9, 20.7)	0.6 (0.2, 0.9)	9.5 (3.2, 14.8)	21.3 (10.2, 49.5)	-3.3 (-15.3, 12.5)	8.0 (-17.5, 16.9)
P value	1.000	0.089	0.085	0.587	0.330	0.017

Data are reported as median (25th, 75th quartiles). TNF‐α: tumor necrosis factor-α; IL: interleukin. The Mann Whitney U test was used for statistical analyses.

## Discussion

A previous study reported an increase in IL‐17A from D1 to D7 and then a decrease after D7 in AIS patients (16). Nevertheless, the longitudinal change of multiple inflammatory cytokines after AIS onset is rarely explored. In the current study, TNF-α, IL-1β, IL-6, IL-8, and IL-17A were increased from baseline to D1 and afterward decreased from D1 to D7, but IL-10 had a reverse tendency in AIS patients, which might be caused by the fact that neuroinflammation dramatically increases after AIS onset and could be alleviative after timely treatment. Thus, pro-inflammatory cytokines (TNF-α, IL-1β, IL-6, IL-8, and IL-17A) increased and the anti-inflammatory cytokine (IL-10) decreased from baseline to D1 and then showed an opposite trend from D1 to D7 (17,18).

Psychological complications are common in cardiac-cerebral vascular disease patients (13,14,19). It has been reported that anxiety, depression, and cognitive impairment rates can range from 20.4 to 39.2%, 26.2 to 31.2%, and 18.6 to 43.2%, respectively, among AIS patients (15,20,21). The occurrence of anxiety, depression, and cognitive impairment in this study were 32.6, 39.4, and 19.4%, respectively, among AIS patients, which was comparable to previous studies. The potential reasons for the relatively high prevalence of these psychological complications in AIS might be due to the fact that AIS causes neuron damage, which could lead to cognitive impairment. In addition, AIS could decrease the quality of life and consequently result in anxiety and depression among these patients (15,22,23).

Inflammatory cytokines are associated with psychological complications among cardiac-cerebral vascular disease patients. For instance, TNF‐α, IL‐1β, IL‐6, and IL‐17A are elevated in coronary heart disease patients with anxiety and depression in comparison to those without these disorders (14). Elevated TNF‐α was correlated to the occurrence of anxiety and cognitive impairment in AIS patients (15). However, the association between inflammatory cytokine longitudinal change after AIS onset at different time points and psychological complications is unclear. In the current study, a positive correlation was found between TNF-α, IL-6, IL-8, and IL-17A after AIS onset and anxiety, depression, or cognitive impairment at different time points. The possible explanations could be that: 1) elevated TNF‐α, IL‐1β, IL‐6, and IL‐17A could regulate indoleamine 2,3-deoxygenation enzyme-1 and consequently modulate 5-hydroxytryptamine, the latter one is involved in the pathogenesis of anxiety and depression (24-26): 2) pro-inflammatory cytokines could accelerate neuronal damage, which further leads to cognitive impairment (22,23).

This study had several limitations that should not be ignored: 1) only first-episode AIS patients were included in this study; thus, the relationship of inflammatory cytokines with anxiety, depression, and cognitive impairment after recurrent AIS onset should be explored in the future; 2) the detailed mechanism of inflammatory cytokines in regulating the pathogenesis of anxiety, depression, and cognitive impairment after AIS onset could also be investigated in another study; 3) the current study lacked follow-up, thus the link between inflammatory cytokines and survival among AIS patients with psychological issues could not be explored; 4) this was a single-center study, leading to unavoidable enrollment bias; 5) mental issues were only assessed once, at discharge.

In conclusion, inflammatory cytokines were dysregulated after AIS onset, and their longitudinal change was correlated with psychological issues among AIS patients, which may be involved in the pathogenesis of these psychological complications. The current discovery may help in the management of these mental disorders in AIS patients.
